# Mechanical ventilation in idiopathic pulmonary fibrosis: a nationwide analysis of ventilator use, outcomes, and resource burden

**DOI:** 10.1186/s12890-017-0426-2

**Published:** 2017-05-22

**Authors:** Joshua J. Mooney, Karina Raimundo, Eunice Chang, Michael S. Broder

**Affiliations:** 1 Stanford University, Stanford, CA USA; 2 Genentech, Inc, South San Francisco, CA USA; 3 Partnership for Health Analytic Research, LLC, Beverly Hills, CA USA

**Keywords:** Mechanical ventilation, Mortality, Outcomes, Cost of illness, Idiopathic pulmonary fibrosis, Noninvasive ventilation

## Abstract

**Background:**

Idiopathic pulmonary fibrosis (IPF) is associated with increased risk of respiratory-related hospitalizations. Studies suggest mechanical ventilation (MV) use in IPF does not improve outcomes and guidelines recommend against its general use. Our objective was to investigate MV use and association with cost and mortality in IPF.

**Methods:**

This retrospective study, using a nationwide sample, included claims with IPF (ICD-9-CM: 516.3) in 2009–2011 and principal respiratory disease diagnosis (ICD-9-CM: 460–519); excluding lung transplant. Regression models were used to determine predictors of MV and association with cost, LOS, and mortality. Domain analysis was used to account for use of subpopulation. Costs were adjusted to 2011. Data on patient severity not available.

**Results:**

Twenty two thousand three hundred fifty non-transplant IPF patients were admitted with principal respiratory disease diagnosis: Mean age 70.0 (SD 13.9), 49.1% female, mean LOS 7.4 (SD 8.2). MV was used in 11.4% of patients with a non-significant decline over time. In regression models, MV was associated with an increased stay of 9.78 days (95% CI 8.38–11.18) and increased cost of $36,583 (95% CI $32,021–41,147). MV users had significantly increased mortality (OR 15.55, 95% CI 12.13–19.95) versus nonusers.

**Conclusions:**

Mechanical ventilation use has not significantly changed over time and is mostly used in younger patients and those admitted for non-IPF respiratory conditions. MV was associated with a 4-fold admission cost increase ($49,924 versus $11,742) and a 7-fold mortality increase (56% versus 7.5%), although patients who receive MV may differ from those who do not. Advances in treatment and decision aids are needed to improve outcomes in IPF.

## Background

Idiopathic pulmonary fibrosis (IPF), a form of interstitial pneumonia, affects 0.5% of US adults over age 65 [1]. The disease is characterized by progressive lung fibrosis [2] and unpredictable episodes of disease worsening, which may lead to hospitalization and frequently death [3–5]. The median survival from diagnosis is 3–5 years [6]. Although two pharmacologic treatments that slow physiologic decline are now available [7, 8], limited options remain for IPF patients hospitalized with respiratory-related symptoms or failure.

Management of respiratory failure in IPF is challenging as patients can develop acute disease episodes that necessitate ventilator support. In select IPF patients, ventilator support can be used as a bridge to lung transplant [9, 10] or could allow for treatment of reversible non-IPF causes of respiratory failure. However, overall outcomes of IPF patients who require non-invasive ventilation or mechanical ventilation (MV) are poor [11–16]. A systematic review [17] summarizing 9 single-center studies reported an 87% in-hospital mortality rate for IPF patients who received MV. Given this evidence, IPF treatment guidelines recommend the majority of IPF patients with respiratory failure not receive MV, and when used should occur after assessing patient-specific goals of care or lung transplant candidacy [10].

While studies have repeatedly demonstrated high mortality with MV, the nationwide pattern of its use in IPF patients has not been well characterized. In this study, we investigated US trends in the use of non-invasive ventilation and MV for IPF, predictors of use, and association with hospital cost, length of stay (LOS), and mortality. We also examined whether MV had a direct effect on mortality, or whether the effect was entirely mediated by the patients’ underlying disease and comorbid conditions.

## Methods

### Design and data sources

We conducted a retrospective cohort study using the Nationwide Inpatient Sample (NIS), the largest publicly available US inpatient database that includes individuals covered by Medicare, Medicaid, or private insurance, as well as the uninsured. Data elements include diagnoses, procedures, demographics, hospital characteristics, payment source, charges, discharge status, LOS, and severity measures [18]. The study used de-identified data and was exempt from institutional review board review.

### Patient Population

We included all hospitalizations from 2009–2011 with claims for IPF (International Classification of Diseases, 9th Revision, Clinical Modification [ICD-9-CM] code 516.3) and a principal diagnosis of respiratory disease (ICD-9-CM: 460–519). Hospital discharge records may contain multiple diagnoses, with the primary cause for admission listed as “principal.” A hospitalization for a patient with IPF admitted with pneumonia as the principal diagnosis would have been included in our study, as pneumonia is a respiratory disease, as would a hospitalization with a principal diagnosis of IPF (also a respiratory disease). An admission with a principal diagnosis of hip fracture would not be included, even if IPF was listed as a secondary diagnosis. Included patients had ≥ 1 inpatient claim with IPF as a discharge diagnosis between 2009–2011. We excluded lung transplant admissions (ICD-9-CM: 33.5×, 33.6).

### Variables

Outcome variables of interest were non-invasive (ICD-9-CM: 93.90) and MV (ICD-9-CM: 96.7×) use, hospital LOS, total inpatient costs, and in-hospital mortality. Other study variables include demographics, primary payer type, hospital characteristics, and all patient refined diagnosis-related group (APR-DRG) severity of illness. APR-DRG assigns patients to severity and mortality subclasses using co-morbidities, age, procedures, and principal diagnosis [19]. We looked for evidence of concomitant acute and chronic pulmonary conditions, including chronic obstructive pulmonary disease (COPD), bacterial pneumonia, and lung cancer. Cardiovascular conditions were identified, including ischemic heart disease, myocardial infarction (MI), congestive heart failure, and pulmonary hypertension. The number of chronic conditions for each patient, calculated using the Chronic Condition Indicator, was reported. This indicator uses 5 digit ICD-9-CM codes to categorize conditions as chronic or not chronic [20]. Admissions were characterized as elective, emergency, urgent, or other non-elective. Discharge disposition was reported as routine, transfer to short-term hospital, transfer to other facilities, home health care, died in hospital, or unknown.

### Statistical analysis

Variables were weighted to represent national estimates and rounded to the nearest integer. NIS reports only charges, so cost-to-charge ratios were used to estimate costs. These ratios are constructed using costs and charge information from hospital reports to CMS. Hospital-specific ratios were used if available; otherwise a weighted group average was used. Costs were adjusted to 2011 US$ using the medical care component of the consumer price index [21]. For categorical variables, Rao-Scott chi-square goodness-of-fit tests adjusting for sampling design were used, relevant p-values reported. We calculated variance using domain analysis to account for subpopulations. Linear regression models were used for LOS and cost, logistic regression models for MV and mortality. Models were adjusted for age, gender, race, principal diagnosis of IPF, lung cancer, selected cardiovascular conditions, hospital region, hospital teaching status, and MV use, as appropriate. Adjusted mean LOS and hospital cost, and adjusted inpatient mortality rate (and 95% confidence intervals) were reported for MV users and nonusers.

Patients with certain characteristics may have a higher risk of inpatient mortality and MV use. To investigate whether MV use was a mediator between clinical characteristics and mortality (rather than directly related), we followed the approach described by Baron and Kenny [22]. We conducted additional regression models to examine the association of clinical conditions/characteristics (the causal variables) on both the MV use (the mediator) and mortality (the outcome variable). Model results were compared to determine whether mediation effects were identifiable. Data transformations and statistical analyses were performed using SAS® version 9.4.

## Results

**Fig. 1:**
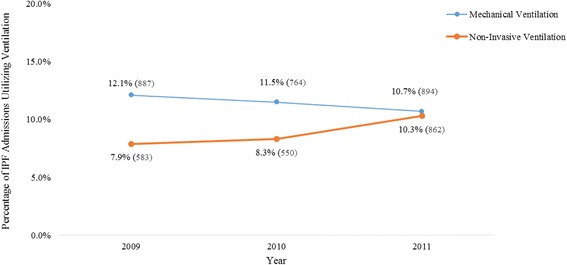
Trend in Ventilation Use in IPF Hospitalizations. The proportion of IPF hospitalizations where mechanical ventilation was used declined each year, going from 12.1% (887) in 2009, to 11.5% (764) in 2010, and 10.7% (894) in 2011 (*p* = 0.578). The use of non-invasive ventilation increased over the same period: 7.9% (583) in 2009, 8.3% (550) in 2010, and 10.3% (862) in 2011 (*p* = 0.112)

From 2009–2011 42,924 IPF patients were admitted to US short-stay hospitals; 23,739 admissions had a principal diagnosis of respiratory disease. The remainder of admissions for these IPF patients were for non-respiratory conditions. After excluding 1,379 lung transplant admissions and 10 with missing age, final sample size was 22,350: 7,346 in 2009, 6,643 in 2010, and 8,362 in 2011. MV was used in 11.4% (2,546) of admissions: 12.1% (887) in 2009, 11.5% (764) in 2010, and 10.7% (894) in 2011 (*p* = 0.578). Non-invasive ventilation was used in 8.9% (1,995) of admissions: 7.9% (583) in 2009, 8.3% (550) in 2010, and 10.3% (862) in 2011 (*p* = 0.112) (Fig. 1).

### Unadjusted analysis

Mean age was 65.9 (+/−0.62) for MV users and 70.5 (+/−0.34) for nonusers (*p* < 0.001). Overall, 49.1% (10,976) of patients were female: 40.2% (1,024) of MV users and 50.3% (9,953) of nonusers (*p* < 0.001). The majority (64.4%, *n* = 14,404) of patients were White, 9.4% Hispanic, 7.6% Black, with no significant difference by MV use. The primary payer was Medicare for 58.9% of admissions at which MV was used, compared to 69.7% where it was not (*p* < 0.001). A principal diagnosis of IPF was present in 31.5% of admissions at which MV was used vs. 44.6% where it was not (*p* < 0.001) (Table 1).

**Table 1:** Patient Demographics, Hospital Characteristics, and Admission Type

	Mean (+/−SE)/no.(%)			*P* Value
	MV *N* = 2,546	No MV *N* = 19,805	All *N* = 22,350	
Age	65.9 (+/−0.62)	70.5 (+/−0.34)	70.0 (+/−0.32)	<.001
Female	1,024 (40.2%)	9,953 (50.3%)	10,976 (49.1%)	<.001
Race				0.657
White	1,639 (64.4%)	12,764 (64.5%)	14,404 (64.4%)	
Black	224 (8.8%)	1,483 (7.5%)	1,707 (7.6%)	
Hispanic	200 (7.8%)	1,910 (9.6%)	2,110 (9.4%)	
Other	129 (5.1%)	999 (5.0%)	1,128 (5.0%)	
Missing	353 (13.9%)	2,649 (13.4%)	3,002 (13.4%)	
Primary payer type				<.001
Medicare	1,499 (58.9%)	13,798 (69.7%)	15,297 (68.4%)	
Medicaid	231 (9.1%)	1,300 (6.6%)	1,531 (6.9%)	
Private (including HMO)	710 (27.9%)	3,880 (19.6%)	4,590 (20.5%)	
Self-pay	41 (1.6%)	408 (2.1%)	448 (2.0%)	
Missing/No charge/Other	65 (2.5%)	420 (2.1%)	484 (2.2%)	
Hospital region				0.845
Northeast	433 (17.0%)	3,465 (17.5%)	3,897 (17.4%)	
Midwest	607 (23.8%)	5,037 (25.4%)	5,644 (25.3%)	
South	1,055 (41.4%)	8,114 (41.0%)	9,169 (41.0%)	
West	452 (17.8%)	3,189 (16.1%)	3,641 (16.3%)	
Teaching hospital	1,332 (52.3%)	8,354 (42.2%)	9,687 (43.3%)	<.001
Bed size				0.022
Small	229 (9.0%)	2,581 (13.0%)	2,811 (12.6%)	
Medium	499 (19.6%)	4,309 (21.8%)	4,807 (21.5%)	
Large	1,771 (69.6%)	12,676 (64.0%)	14,447 (64.6%)	
Missing	47 (1.8%)	239 (1.2%)	286 (1.3%)	
Evidence of ED services^a^	1,650 (64.8%)	13,262 (67.0%)	14,912 (66.7%)	0.363
Principal diagnosis of IPF	802 (31.5%)	8,823 (44.6%)	9,626 (43.1%)	<.001
Elective admission^b^	361 (14.2%)	3,152 (15.9%)	3,512 (15.7%)	0.307

^a^Defined by NIS as having either an ED revenue code, charge, CPT procedure code, or admission source, or being on a state-defined ED record

^b^Defined by NIS as admission other than emergency, urgent, newborn, delivery, trauma center, or other-non elective

ICD-9-CM diagnoses of pneumonia (49.2% vs. 37.1%, *p* <0.001) and MI (10.5% vs. 5.4%, *p* <0.001) were more common in patients requiring MV, while COPD (28.9% vs. 39.4%, *p* < 0.001) was less common. As is the case for all diagnoses in this study, these conditions were not confirmed clinically. MV users had significantly fewer chronic conditions (4.2 vs. 4.3, *p* <0.001) (Table 2). Patients who used MV had longer hospital stays (16.5 days [+/−0.73] vs. 6.2 [+/−0.10], *p* < 0.001), were more likely to have died in the hospital (55.3% vs. 8.8%) and less likely to have a routine home discharge (9.3% vs. 51.2%) (*p* < 0.001). Costs ($49,924 vs. $11,742, *p* < 0.001) were higher in MV users compared to nonusers (Table 3).

**Table 2:** Patient Clinical Characteristics and Treatment

	Mean (+/−SE)/no.(%)			*P* Value
	MV *N* = 2,546	No MV *N* = 19,805	All *N* = 22,350	
No. of chronic conditions	4.2 (+/−0.06)	4.3 (+/−0.03)	4.3 (+/−0.03)	<.001
Chronic obstructive pulmonary disease	736 (28.9%)	7,800 (39.4%)	8,535 (38.2%)	<.001
Bacterial pneumonia	1,252 (49.2%)	7,352 (37.1%)	8,604 (38.5%)	<.001
Lung cancer	59 (2.3%)	348 (1.8%)	407 (1.8%)	0.380
Cardiovascular conditions	1,229 (48.3%)	8,835 (44.6%)	10,063 (45.0%)	0.137
Ischemic heart disease	717 (28.2%)	5,622 (28.4%)	6,339 (28.4%)	0.913
Myocardial infarction	267 (10.5%)	1,078 (5.4%)	1,345 (6.0%)	<.001
Congestive heart failure	793 (31.1%)	5,427 (27.4%)	6,219 (27.8%)	0.119
Pulmonary hypertension	19 (0.8%)	65 (0.3%)	84 (0.4%)	0.146
APR-DRG severity of illness				<.001
Minor loss of function	5 (0.2%)	443 (2.2%)	447 (2.0%)	
Moderate loss of function	16 (0.6%)	5,042 (25.5%)	5,058 (22.6%)	
Major loss of function	341 (13.4%)	10,197 (51.5%)	10,538 (47.1%)	
Extreme loss of function	2,184 (85.8%)	4,123 (20.8%)	6,307 (28.2%)

**Table 3:** Patient Discharge Status, LOS, and Total Costs

	Mean (+/−SE)/no.(%)			*P* Value
	MV Use *N* = 2,546	No MV Use *N* = 19,805	All *N* = 22,350	
Discharge status				<.001
Routine	236 (9.3%)	10,131 (51.2%)	10,367 (46.4%)	
Transfer to short-term hospital	175 (6.9%)	548 (2.8%)	724 (3.2%)	
Transfer to other facilities	531 (20.8%)	3,353 (16.9%)	3,883 (17.4%)	
Home health care	181 (7.1%)	3,937 (19.9%)	4,118 (18.4%)	
Died in hospital	1,408 (55.3%)	1,738 (8.8%)	3,146 (14.1%)	
Other^a^	15 (0.6%)	98 (0.5%)	112 (0.5%)	
Days of stay (among all IPF patients)	16.5 (+/−0.73)	6.2 (+/−0.10)	7.4 (+/−0.15)	<.001
Died in hospital	1,408 (55.3%)	1,738 (8.8%)	3,146 (14.1%)	<.001
Total inpatient costs (2011 US$)	$49,924 (+/−2,490)	$11,742 (+/−390)	$16,042 (+/−631)	<.001

^a^Against medical advice, discharged alive, or destination unknown

### Adjusted analysis

**Fig. 2:**
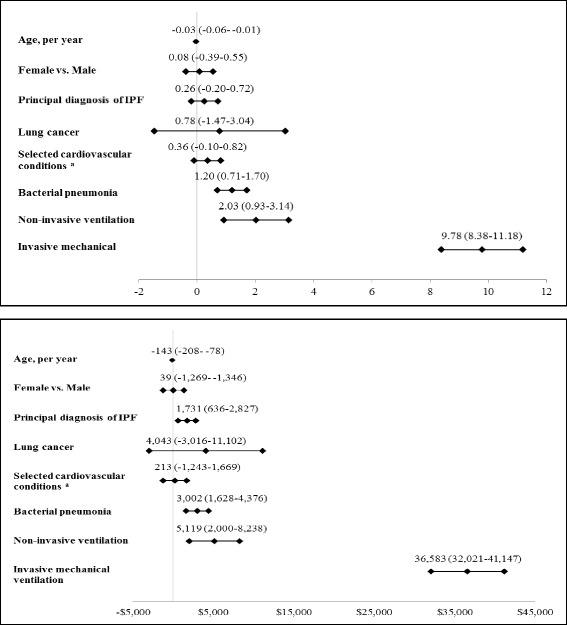
Linear Regression Model for LOS and Costs. Age, bacterial pneumonia, and use of mechanical ventilation were statistically significantly (*p* < 0.001) associated with cost and LOS. Admission with a principal diagnosis of IPF was significantly associated with cost but not LOS. Use of mechanical ventilation had the largest effect on LOS and cost, with an increase of 9.78 days [95% CI: 8.38 - 11.18] and $36,583 [32,021 – 41,147] respectively. Non-invasive ventilation was associated with an increase of 2.03 days [0.93 – 3.14] in LOS and $5,119 [2,000 – 8,238] in cost. Point estimates and 95% CI for LOS and cost are adjusted for all listed variables. *CI* Confidence interval; ^a^ Ischemic heart disease, myocardial infarction, and congestive heart failure

MV was associated with an adjusted LOS of 16.1 days (95% CI: 15; 17.5) versus 6.3 days (95% CI: 6; 6.5) for nonusers. The adjusted cost associated with MV was $48,772 (95% CI: 43,979; 53,565) versus $11,861 (95% CI: 11,292; 12,431) for nonusers. The adjusted in-hospital death rate for MV users and nonusers was 55.7% (95% CI: 50.3; 61.0) and 7.5% (95% CI: 6.6; 8.4) (Table 4). Each year of increased age was associated with shorter LOS (−0.03; 95% CI: −0.06; −0.01) and lower cost ($-143; 95% CI: −208; −78) but greater in-hospital death (OR 1.02; 95% CI: 1.01; 1.03). The use of non-invasive ventilation was associated with increased LOS (2.03 days; 95% CI: 0.93; 3.14), cost ($5,119; 95% CI: 2,000; 8,238) and death (OR 4.77; 95% CI: 3.48; 6.55) (Fig. 2). A principal diagnosis of IPF was associated with increased cost ($1,731; 95%CI: 636; 2,827) and death (OR 1.78; 95% CI: 1.42; 2.24) but no change in LOS (Fig. 2).

**Table 4:** Adjusted LOS, Inpatient Costs, and In-Hospital Death Rate^a^

IMV Use	Adjusted^a^ in-Hospital Death Rate (95% CI)	Adjusted^a^ OR (95% CI)	*P* Value
Yes	55.7% (50.3 – 61.0)	15.55 (12.13 – 19.95)	<.001
No	7.5% (6.6 – 8.4)	ref	

*CI* Confidence interval; *OR *Odds ratio

^a^Adjusted by age, gender, race, hospital region, teaching hospital, principal diagnosis of IPF, lung cancer, selected cardiovascular conditions (ischemic heart disease, myocardial infarction, and congestive heart failure), and non-invasive ventilatio4n use

To investigate the association among clinical conditions/characteristics, MV use, and inpatient mortality, we conducted two additional logistic regression models. In the model for risk of MV, we controlled for patient and hospital characteristics. In this model, decreasing age (OR 0.97, 95% CI: 0.97; 0.98), female gender (OR 0.68, 95% CI: 0.55; 0.85), Hispanic ethnicity (OR 0.66, 95% CI: 0.45; 0.97) and principal diagnosis of IPF (OR 0.60, 95% CI: 0.48; 0.76) were associated with a lower risk of MV. Cardiovascular conditions (OR 1.34, 95% CI: 1.08; 1.65; *p* = 0.007), bacterial pneumonia (OR 1.55, 95% CI: 1.27; 1.90; *p* <0.001), and teaching hospital admission (OR 1.58, 95% CI 1.26; 1.98; *p* < 0.001) were associated with higher risk of MV. In the model for in-hospital death that excluded MV as a predictor, female gender was associated with a lower risk of death (OR 0.62, 95% CI: 0.52; 0.74; *p* < 0.001), whereas principal diagnosis of IPF (OR 1.26, 95% CI: 1.03; 1.55; *p* = 0.026), teaching hospital admission (OR 1.37, 95% CI 1.11; 1.69; *p* = 0.003), cardiovascular conditions (OR 1.26, 95% CI: 1.04; 1.51; *p* = 0.017), and bacterial pneumonia (OR 1.42. 95% CI: 1.18; 1.71; *p* < 0.001) were associated with increased risk (Table 5).

**Table 5:** Logistic Regression Model Results for Risk of Mechanical Ventilation and Death

Parameter	Logistic Regression Model			
	Risk of In-Hospital Death		Risk of Invasive Mechanical Ventilation	
	OR (95% CI)	*P* Value	OR (95% CI)	*P* Value
Age, per year	1.00 (1.00 – 1.01)	0.562	0.97 (0.97 – 0.98)	<.001
Female vs. Male	0.62 (0.52 – 0.74)	<.001	0.68 (0.55 – 0.85)	<.001
Race				
Black vs. White	0.70 (0.47 – 1.04)	0.081	0.98 (0.68 – 1.39)	0.896
Hispanic vs. White	0.80 (0.56 – 1.15)	0.235	0.66 (0.45 – 0.97)	0.036
Other vs. White	1.05 (0.68 – 1.62)	0.836	0.86 (0.52 – 1.42)	0.547
Missing vs. White	1.09 (0.78 – 1.52)	0.627	0.99 (0.71 – 1.39)	0.966
Hospital region				
Northeast vs. West	0.95 (0.65 – 1.37)	0.776	0.75 (0.50 – 1.11)	0.151
Midwest vs. West	0.85 (0.60 – 1.20)	0.362	0.77 (0.53 – 1.11)	0.161
South vs. West	0.92 (0.68 – 1.25)	0.605	0.82 (0.59 – 1.12)	0.207
Teaching hospital	1.37 (1.11 – 1.69)	0.003	1.58 (1.26 – 1.98)	<.001
Principal diagnosis of IPF	1.26 (1.03 – 1.55)	0.026	0.60 (0.48 – 0.76)	<.001
Lung cancer	1.71 (0.99 – 2.94)	0.053	1.12 (0.57 – 2.20)	0.750
Selected cardiovascular conditions ^a^	1.26 (1.04 – 1.51)	0.017	1.34 (1.08 – 1.65)	0.007
Bacterial pneumonia	1.42 (1.18 – 1.71)	<.001	1.55 (1.27 – 1.90)	<.001

*OR* Odds ratio, *CI* Confidence interval

^a^Ischemic heart disease, myocardial infarction, and congestive heart failure

## Discussion

Our study of IPF patients admitted to a nationwide sample of acute care hospitals found 11-12% of IPF patients admitted with a respiratory condition used MV, with no significant change from 2009–2011. Younger, male patients with fewer comorbidities and/or with a non-IPF principal diagnosis (e.g., pneumonia) were more likely to use MV. MV was associated with nearly 10-day longer hospital stays, $37,000 higher cost, and a more than 7-fold increase in mortality (56% versus 7.5%). Less than 10% of patients who used MV were discharged home routinely, compared to more than half of nonusers. Non-invasive ventilation was associated with increased LOS and cost, although to a lesser extent than MV.

The unchanging nationwide use of MV over time, despite IPF treatment guidelines conditionally recommending against MV use, reflects the limited options available to clinicians treating acute worsening of IPF and the difficulty of advance care planning in IPF. As acute worsening leading to respiratory failure can occur quickly and unexpectedly, MV can provide time to evaluate for possible treatable conditions, to assess patient preferences and/or to support gas-exchange while awaiting lung transplant. Lung transplantation remains the only curative and life-prolonging option for select patients with advanced IPF and respiratory failure. Notably, IPF patients who received MV were younger with fewer chronic medical conditions, more often admitted at a teaching hospital, and more frequently coded with a non-IPF principal respiratory diagnosis (e.g., pneumonia). This suggests a nationwide preference for MV use in younger, somewhat healthier, IPF patients or in those with a clinical suspicion of a reversible condition. Possible explanations for this finding are that younger patients with less chronic comorbidity may be potential lung transplant candidates or clinicians may feel compelled to offer them a trial of ventilator support. We cannot ascertain from the data if patients were awaiting transplant or later transferred for transplant evaluation.

The overall economic and health care burden of IPF is well-recognized [23–27]. This study uniquely highlights the burden associated with MV use in IPF, while reinforcing with nationwide data the poor outcomes reported in prior smaller studies. Hospital cost was more than 4-fold greater and mortality 7-fold greater in IPF patients hospitalized with a respiratory problem requiring MV. While in-hospital mortality (55.3%) was lower than previously reported, this underestimates mortality as a significant number of patients were transferred to short-term hospitals (6.9%) or other facilities (20.8%) where their final vital status is unknown. Only 16.4% of MV users were discharged home. The high mortality and economic burden associated with MV in IPF stresses the need to improve the quality of medical care for IPF patients, including advances in prevention, treatment, and patient-clinician shared decision-making. While recently approved pharmacologic therapies slow disease progression and may reduce acute exacerbations [7, 8, 28], the course of IPF remains unpredictable. Therefore, early patient-centered discussions on treatment expectations, appropriate referrals for transplant and/or palliative care, and coordination of care across providers, remain integral to honoring patients’ values while ensuring high value care. IPF-specific decision aids are needed to help guide patients.

Some conditions that lead to MV may themselves be associated with greater mortality, confounding the interpretation of our findings. We used a method similar to that of Baron and Kenny [22] to test whether MV simply mediated the mortality effects of other variables. Our results suggest that this was not true of a principal IPF diagnosis, as it remains associated with mortality in the models both with and without MV use. Both cardiovascular conditions and bacterial pneumonia had statistically significant effects in the model that did not include MV, and smaller, not statistically significant, effects in the model that included it. This suggests the association between these characteristics and in-hospital death results, at least in part, from their association with MV, which independently increases the risk of death. However, some residual confounding by indication likely still exists.

This study has limitations. First, there is debate on how to identify IPF patients using claims data. The ICD-9-CM code we used has been used before in several publications [1, 6, 23, 24], however a recent validation study (not in the NIS) found it had a positive predictive value of 30-60% [29]. While less than desirable, this positive predictive value is within the range reported in study of ICD-9-CM codes for 32 conditions (median 80.7%, mean 77%, range 23-100%) [30]. Similarly, none of the conditions we identified (e.g., COPD) were confirmed clinically. Identification relied on ICD-9-CM codes, which are designed and used primarily for billing. Our study was further limited in that the NIS does not allow patients to be followed through subsequent outpatient care, repeat hospitalizations, or transfer to other facilities. We could not determine whether a subject died, received a transplant, or was discharged home after being transferred. The study relied on secondary data collected at discharge for administrative purposes, so no clinical information, including disease severity, was available. We could not determine whether medical conditions were present on admission or developed during hospitalization, nor could we determine the order in which diagnoses were made or treatments were given. Further, less severe comorbid conditions common to patients with IPF (e.g., obesity and gastroesophageal reflux) may be undercoded. Finally, as in prior studies [23, 24] we excluded transplant-related expenditures. This exclusion allows for a close look at direct costs of IPF-related care, but underestimates the complete cost of IPF.

A strength of the study is the use of the Nationwide Inpatient Sample, which was designed to inform policy decisions regarding health and health care at national and regional levels. Previous evaluations of IPF MV use and cost have been limited to specific centers or populations (e.g., Medicare and select private insurers) and their findings may be less generalizable. The NIS includes patients with Medicare, Medicaid, and private insurance, as well as the uninsured, making this dataset the best way to produce estimates valid for the overall US population.

## Conclusion

In a nationwide sample of IPF patients, MV was used in 11-12% of those hospitalized due to a respiratory diagnosis with no significant change in its use over time. Mechanical ventilation was more frequent in younger male IPF patients, those admitted at teaching hospitals, and those with fewer chronic medical conditions or a non-IPF respiratory diagnosis. Its use was associated with a 4-fold increase in admission cost ($49,924 compared to $11,742) and a 7-fold increase in admission mortality (56% compared to 7.5%). Further advances in IPF treatment and development of IPF-specific decision aids are needed to improve the resource burden, outcomes, and use of MV in IPF.

## Abbreviation

APR-DRG: All patient refined diagnosis-related group
CI: Confidence interval
COPD: Chronic obstructive pulmonary disease
CPT: Current procedural terminology
ED: Emergency department
ICD-9-CM: International classification of diseases, 9th revision, clinical modification
IPF: Idiopathic pulmonary fibrosis
LOS: Length of stay
MI: Myocardial infarction
MV: Mechanical ventilation
NIS: Nationwide inpatient sample
OR: Odds ratio
Revision: Clinical modification
SD: Standard deviation
